# Consequences of the association between HTLV-1 and tuberculosis

**DOI:** 10.1186/1742-4690-12-S1-P77

**Published:** 2015-08-28

**Authors:** Maria de Lourdes Santana Bastos, Yuri Neves, Natália Carvalho, Anselmo Souza, Abraão Neto, Isadora Siqueira, Silvane Santos, Edgar M Carvalho

**Affiliations:** 1 Serviço de Imunologia - Hospital Universitário Professor Edgard Santos, Salvador, Bahia, Brazil; 2 Centro de Pesquisa Gonçalo Muniz – FIOCRUZ, Salvador, Bahia, Brazil

## 

HTLV-1 modifies the immune response and clinical presentation of bacterial, fungal and helminthic infections. There are also evidences that helminthic infections may modify disease expression related to HTLV-1, increasing the occurrence of Adult T cell leukemia (ATL) and decreasing the development of HTLV-1 associated myelopathy or tropical spastic paraparesis (HAM/TSP). Recently it has been shown that HTLV-1 increases susceptibility for tuberculosis (TB) but the consequences of this co-infection in the clinical course of TB and HTLV-1 infection is not so clear. The aim of this study was to evaluate if TB influences neurologic disease in HTLV-1 infected subjects and to evaluate sequels of TB in HTLV-1 infected individuals. This is a nested case control study with the participation of 32 patients with previous diagnosis of TB and positivity sorology for HTLV-1 and 32 controls with HTLV-1 without TB paired by age, gender and time of admission in the clinic. Of the 32 patients with TB 29 had pulmonary TB, one had pleural TB, one laryngeal TB and the other lymphonode TB. Only 2 (6,2%) developed a second episode of TB. Among those who have been treated for TB 8 (25%) patients complained of dyspnea, 3 without evidence of pulmonary disease, 3 cases had chronic obstructive pulmonary disease (COPD) and two cases had bronchial overactivity. Regarding HTLV-1 associated disease probable HAM/TSP was diagnosed in 4 (12,5%) of the TB patients and in 6 (18,7%) of the patients who only have HTLV-1 infection (P > 0.05). Definitive HAM/TSP was diagnosed in 10 (31,2%) of the co-infected patients with HTLV-1 and TB and in 4 (12,5%) in the group who only had HTLV-1 (P < 0.05). While HTLV-1 appears to not influence the severity of Micobacterium tuberculosis infection TB was highly associated with HAM/TSP.

